# The class I myosin MYO1D binds to lipid and protects against colitis

**DOI:** 10.1242/dmm.035923

**Published:** 2018-09-27

**Authors:** William McAlpine, Kuan-wen Wang, Jin Huk Choi, Miguel San Miguel, Sarah Grace McAlpine, Jamie Russell, Sara Ludwig, Xiaohong Li, Miao Tang, Xiaoming Zhan, Mihwa Choi, Tao Wang, Chun Hui Bu, Anne R. Murray, Eva Marie Y. Moresco, Emre E. Turer, Bruce Beutler

**Affiliations:** 1 Center for the Genetics of Host Defense, University of Texas Southwestern Medical Center, Dallas, TX 75390-8505, USA; 2 Department of Internal Medicine, Division of Gastroenterology, University of Texas Southwestern Medical Center, Dallas, TX 75390-8505 USA; 3 Quantitative Biomedical Research Center, Department of Clinical Science, University of Texas Southwestern Medical Center, Dallas, TX 75390, USA

**Keywords:** Dextran sodium sulfate, N-ethyl-N-nitrosourea, Inflammatory bowel disease

## Abstract

Myosin ID (MYO1D) is a member of the class I myosin family. We screened 48,649 third generation (G3) germline mutant mice derived from N-ethyl-N-nitrosourea-mutagenized grandsires for intestinal homeostasis abnormalities after oral administration of dextran sodium sulfate (DSS). We found and validated mutations in *Myo1d* as a cause of increased susceptibility to DSS-induced colitis. MYO1D is produced in the intestinal epithelium, and the colitis phenotype is dependent on the nonhematopoietic compartment of the mouse. Moreover, MYO1D appears to couple cytoskeletal elements to lipid in an ATP-dependent manner. These findings demonstrate that MYO1D is needed to maintain epithelial integrity and protect against DSS-induced colitis.

## Abstract (precis)

**Summary:** Using random germline mutagenesis and screening of mice, we determined that loss of MYO1D function in nonhematopoietic tissues renders mice susceptible to colitis induced by dextran sodium sulfate challenge.

## INTRODUCTION

Inflammatory bowel diseases (IBDs) are characterized by a state of chronic gut inflammation, believed to have both genetic and environmental etiologies. Although some monogenic forms of IBD have been described (Uhlig, 2013), the genetic component remains unknown in the great majority of cases. Genome-wide association studies (GWAS) have implicated over 200 loci associated with IBD; however, these studies are limited in their ability to determine causative genes because of linkage disequilibrium in the human genome, and they establish correlations rather than cause and effect (Beaudoin et al., 2013; Jostins et al., 2012). We and others (Abraham and Cho, 2009; Maloy and Powrie, 2011) posit that on many occasions, a departure from homeostasis occurs with a breach of the epithelial barrier. Most individuals rapidly repair the breach, eliminate or contain microbes that have entered the lamina propria and limit the inflammatory response, but some are unable to do so. Such individuals can be said to have defective intestinal homeostatic mechanisms.

To identify proteins with nonredundant function in maintaining homeostasis once a breach in the epithelium has occurred, we developed an environmentally sensitized screen in mice (Brandl and Beutler, 2012; Brandl et al., 2009, 2012; Turer et al., 2017). Orally administered dextran sodium sulfate (DSS) damages the intestinal mucosa, creating conditions in which mechanisms mediating a return to homeostasis are tested. In normal C57BL/6J mice, all the requisite mechanisms are intact. We used random germline mutagenesis to damage or destroy genes and determined that mutations in *Myo1d* confer hypersensitivity to low-dose DSS (Brandl and Beutler, 2012; Brandl et al., 2009, 2012; Turer et al., 2017). In this study, we report that MYO1D is necessary for the maintenance of intestinal homeostasis and provide an initial phenotypic characterization of the colitis phenotype caused by loss of MYO1D function.

## RESULTS

### Recessive mutations in *Myo1d* lead to DSS-induced colitis susceptibility

**Fig. 1.**
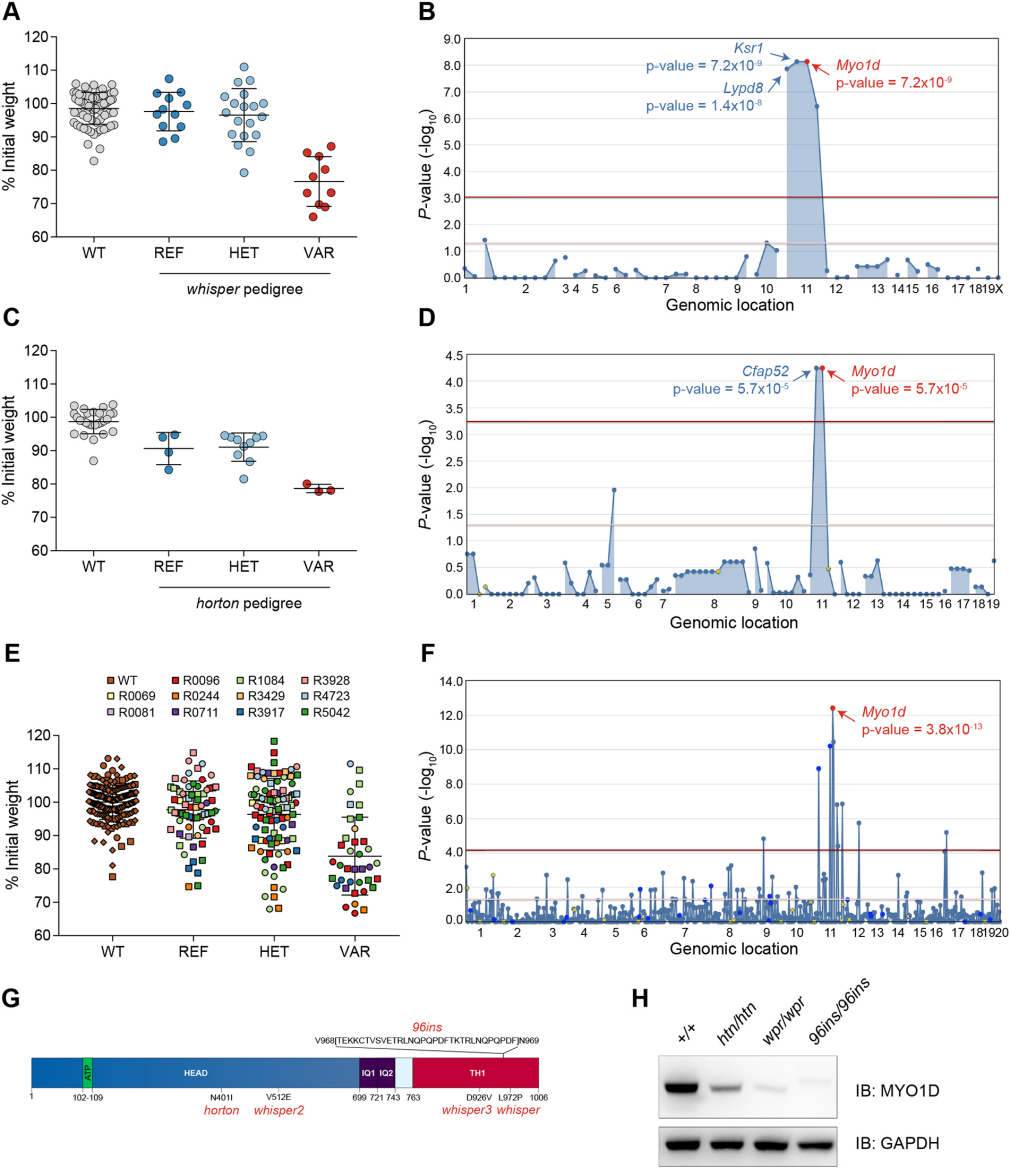
**Mapping of the *Myo1d* mutations in *whisper* and *horton*.** (A) Percentage of initial body weight on day 10 of DSS treatment plotted versus genotype [WT, C57BL/6J (*n*=74); REF, *Myo1d^+/+^* (*n*=12); HET, *Myo1d^+/wpr^* (*n*=19); VAR, *Myo1d^wpr/wpr^* (*n*=10)]. (B) Manhattan plot showing *P*-values of association between the *whisper* phenotype and mutations identified in the *whisper* pedigree, calculated using a recessive model of inheritance. The −log_10_ *P*-values (*y*-axis) are plotted versus the chromosomal positions of the mutations (*x*-axis). Horizontal red and purple lines represent thresholds of *P*=0.05 with or without Bonferroni correction, respectively. *P*-values for linkage of mutations in *Myo1d*, *Ksr1* and *Lypd8* with the *whisper* phenotype are indicated. (C) Body weight data on day 7 of DSS treatment graphed as in A [WT, C57BL/6J (*n*=27); REF, *Myo1d^+/+^* (*n*=4), HET, *Myo1d^+/htn^* (*n*=10), VAR, *Myo1d^htn/htn^* (*n*=3)]. (D) Manhattan plot generated as in B for the *horton* phenotype. *P*-values for linkage of mutations in *Myo1d* and *Cfap52* with the *horton* phenotype are indicated. (E) Percentage of initial body weight plotted versus *Myo1d* genotype for mice from 11 unrelated pedigrees (R0069, R0081, R0096, R0244, R0711, R1084, R3429, R3917, R3928, R4723, R5042) with distinct *Myo1d* mutations. (F) Manhattan plot generated as in B using body weight and genotype data from E. The *P*-value for linkage of *Myo1d* mutations with the DSS-induced weight loss phenotype is indicated. (G) Protein domain organization of mouse MYO1D. The locations of the *horton*, *whisper*, *whisper2* and *whisper3* mutations are shown. The *horton* mutation is an asparagine (N) to isoleucine (I) substitution at amino acid 401 in the head domain (N401I). The *whisper* mutation is a leucine (L) to proline (P) substitution at position 972 (L972P) in the TH1 domain. The *whisper2* mutation is a valine (V) to glutamic acid (E) substitution at position 512 (V512E) in the head domain. The *whisper3* mutation is an aspartic acid (D) to a valine (V) substitution at 926 (D926V). (H) Representative immunoblot showing MYO1D levels in colon epithelium isolated from wild-type, *horton*, *whisper* and *Myo1d^96ins/96ins^* mice. GAPDH was used as a loading control (*n*=3 samples from each genotype). ATP, adenosine 5′-triphosphate binding site; HET, heterozygous for mutant and reference alleles; IB, immunoblot; IQ, IQ calmodulin-binding motifs (IQXXXRGXXXR/K, where X is any amino acid); REF, homozygous for reference allele; TH1, tail homology-1 domain; VAR, homozygous for mutant allele; WT, wild type; 96ins, 96 bp insertion.

N-ethyl-N-nitrosourea (ENU) and a previously described inbreeding scheme were used to generate mice with random mutations in the heterozygous and homozygous state (Wang et al., 2015). We subjected 48,649 third generation (G3) mice from 1870 pedigrees to 1.3-1.5% DSS in their drinking water, and body weights of the mice were recorded daily. Susceptibility to DSS-induced colitis, manifested as body weight loss on day 7 or 10 of DSS treatment relative to initial weight, was detected in two pedigrees, R0096 and R0244. The colitis phenotype in both pedigrees mapped to damaging *Myo1d* alleles using a recessive model of inheritance. The phenotypes were designated *whisper* (*wpr*; pedigree R0096) and *horton* (*htn*; pedigree R0244). The *whisper* phenotype was mapped to mutations in three genes on chromosome 11: kinase suppressor of Ras-1 (*Ksr1*) (*P*=7.2×10^−9^), *Myo1d* (*P*=7.2×10^−9^) and Ly6/PLAUR domain-containing 8 (*Lypd8*) (*P*=1.4×10^−8^) (Fig. 1A,B). The *horton* phenotype was mapped to mutations in two genes on chromosome 11: cilia- and flagella-associated protein 52 (*Cfap52*) and *Myo1d* (*P*=5.7×10^−5^) (Fig. 1C,D). Both *Myo1d* mutations were missense errors predicted to be ‘probably damaging’ by PolyPhen-2 (Adzhubei et al., 2010), with scores of 0.986 and 1.000 for the *whisper* and *horton* alleles, respectively. In all, 11 ENU-induced alleles of *Myo1d* were tested, and five of them appear to be damaging based on their phenotypic effects (Fig. 1E,F). MYO1D is a member of the class I myosin family and contains an N-terminal motor domain and a C-terminal tail homology (TH1) domain. The *whisper* allele encodes a Leu972Pro substitution in the TH1 domain, and the *horton* allele encodes an Asn401Ile substitution in the motor domain (Fig. 1G). Immunoblotting of epithelial extracts from *Myo1d^wpr/wpr^* and *Myo1d^htn/htn^* mice showed reduced levels of MYO1D protein (Fig. 1H), suggesting that both mutations affect protein stability.

**Fig. 2.**
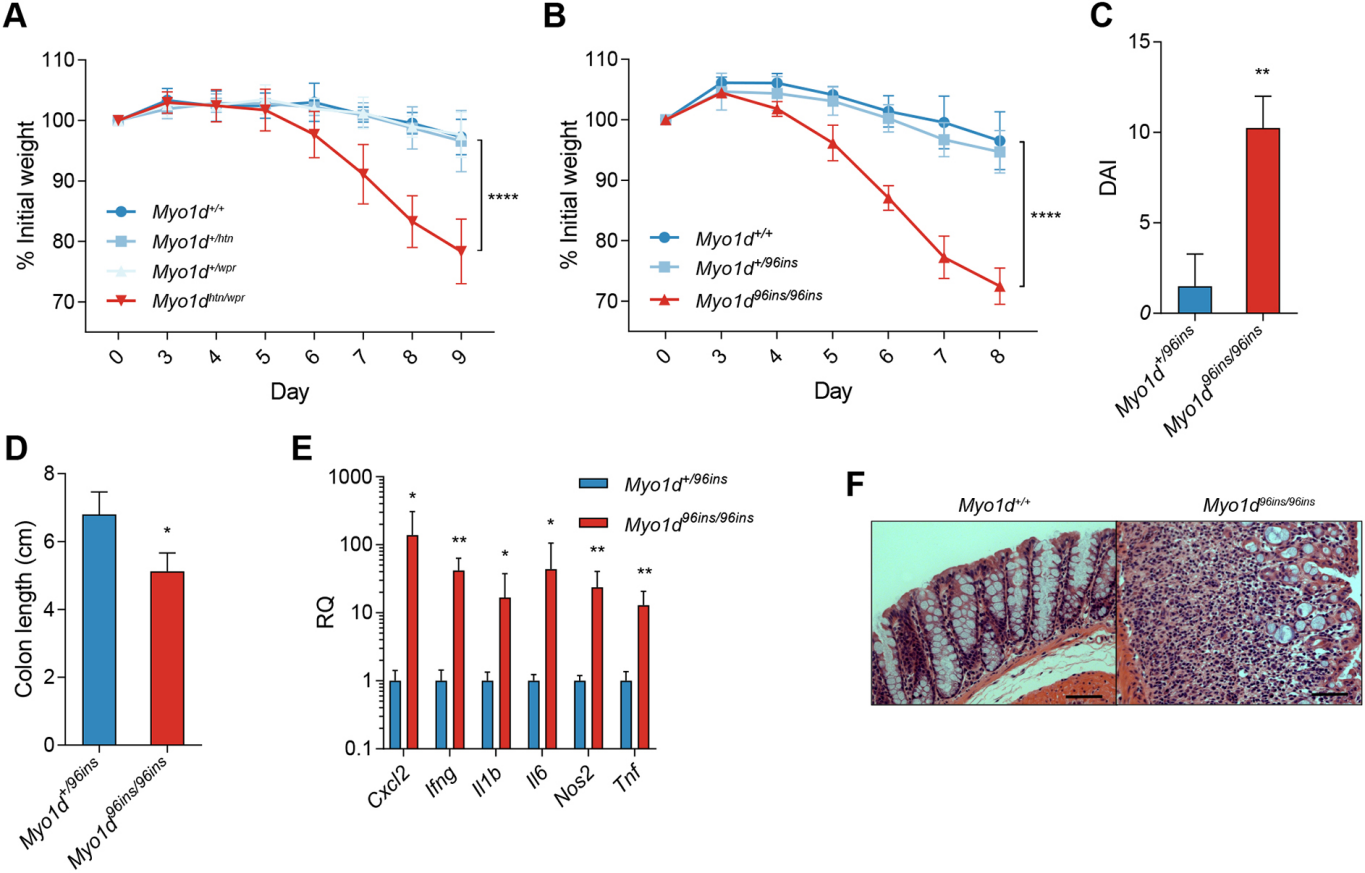
**Validation that *Myo1d* mutations cause a colitis phenotype.** (A) Weight loss analysis of *Myo1d^+/+^*, *Myo1d^+/htn^*, *Myo1d^+/wpr^* and *Myo1d^htn/wpr^* after 1.4% DSS treatment (*n*=4 for all groups). (B) Weight loss analysis of *Myo1d^+/+^* (*n*=5), *Myo1d^+/96ins^* mice (*n*=12) and *Myo1d^96ins/96ins^* mice (*n*=6) after 1.4% DSS treatment. (C,D) Disease activity index (DAI) (C) and colon length (cm) (D) of *Myo1d^+/96ins^* (*n=8*) and *Myo1d^96ins/96ins^* (*n=8*) mice 8 days after DSS challenge. (E) Relative mRNA expression levels of inflammatory cytokines *Cxcl2*, *Ifng*, *Il1b*, *Il6*, *Nos*2 and *Tnf* in the distal colon of *Myo1d^+/96ins^* (*n*=6) and *Myo1d^96ins/96ins^* (*n*=6) mice after 4 days of 1.4% DSS. (F) Representative Hematoxylin and Eosin staining of *Myo1d^96ins/96ins^* colons after 7 days of DSS treatment. Scale bars: 50 μm. For all experiments, 1.4% DSS was administered to mice. Each experiment was performed a minimum of three times. Data are expressed as means±s.d. and significance was determined by two-way analysis of variance (ANOVA) with Dunnett's multiple comparisons (A,B) or unpaired Student’s *t*-test (C-E) (*P<0.05, **P<0.01, ****P<0.0001).

To verify that *Myo1d* mutations were causative of phenotype in these pedigrees, we crossed *horton* heterozygotes (*Myo1d^+/htn^*) with *whisper* heterozygotes (*Myo1d^+/wpr^*) to generate *Myo1d* compound heterozygotes with simple heterozygosity for all other ENU-induced mutations. *Myo1d^htn/wpr^* mice remained susceptible to DSS challenge with 20% weight loss by day 9 of treatment, validating causation (Fig. 2A). To further confirm that mutations in *Myo1d* result in a DSS-induced colitis susceptibility phenotype, CRISPR/Cas9-mediated targeting was used to generate a 96 bp insertion (96ins) in *Myo1d*, which resulted in the in-frame addition of 32 amino acids in the TH1 domain of the MYO1D protein (Fig. 1G,H). During low-dose DSS treatment, *Myo1d^96ins/96ins^* mice lost ∼10% of their body weight by day 6, and more than 25% of their body weight by day 8 (Fig. 2B). Weight loss was accompanied by higher disease activity index (DAI), colonic shortening and increased expression of proinflammatory genes (*Cxcl2*, *Ifng*, *Il6*, *Il1b*, *Nos2* and *Tnf*) in distal colons of *Myo1d^96ins/96ins^* mice (Fig. 2C-E)*.* Colons from DSS-treated *Myo1d^96ins/96ins^* mice showed marked histopathological changes characterized by infiltration of lymphocytes and loss of crypt architecture (Fig. 2F). DSS treatment was lethal for *Myo1d^96ins/96ins^* mice by day 10.

### *Myo1d* mutations sensitize to DSS in a hematopoietic extrinsic manner

**Fig. 3.**
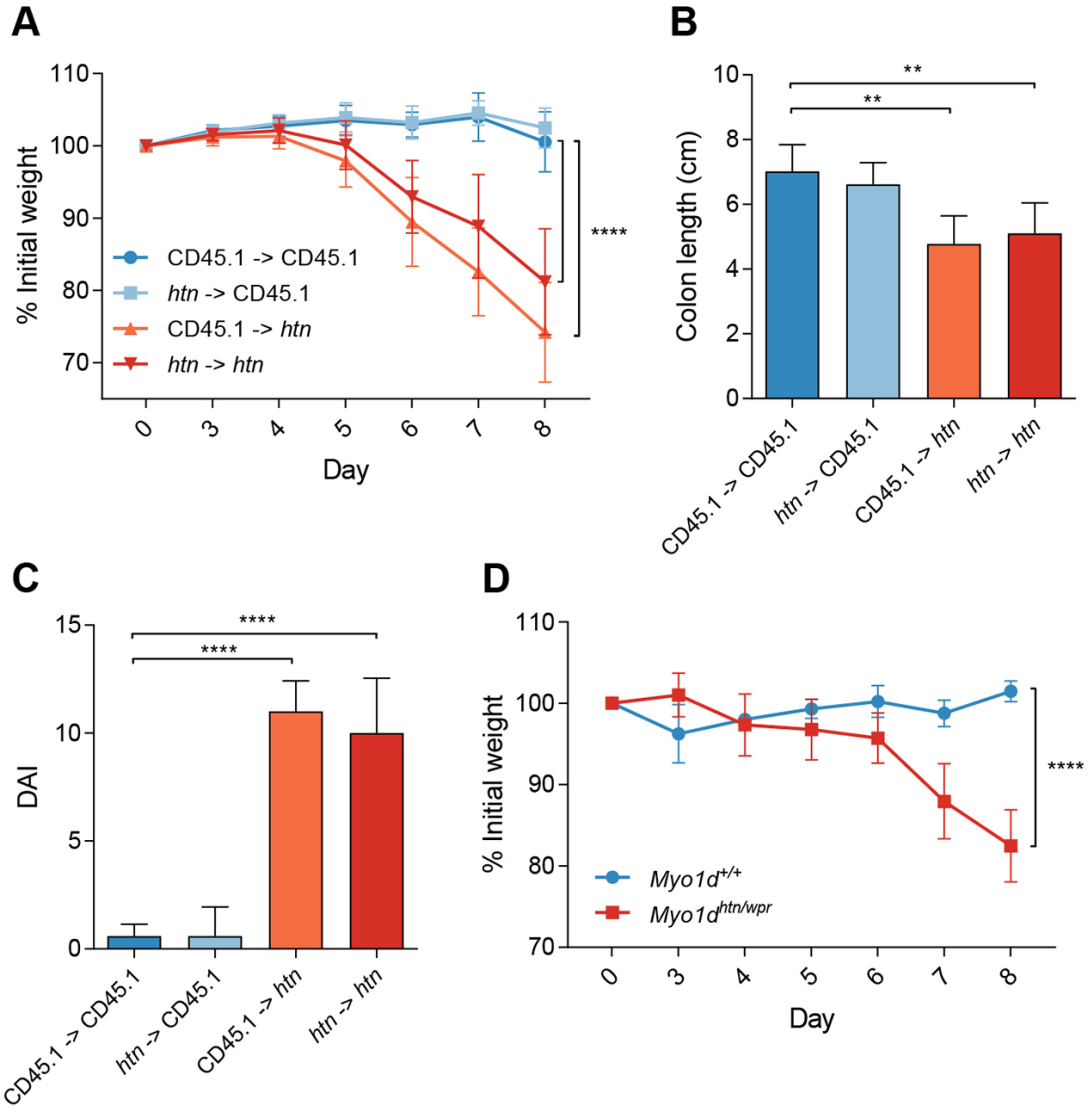
**Nonhematopoietic defects contribute to colitis.** (A-C) Bone marrow chimeras were generated, and percentage of initial body weight (A), colon length (B) and DAI (C) were determined after 1.4% DSS administration (*n*=5 for all groups). (D) Percentage of initial body weight of *Myo1d^+/+^* (*n*=4) or *Myo1d^htn/wpr^* (*n*=5) mice after daily oral gavage with 2.5 g/kg DSS. Data are expressed as means±s.d. and significance was determined by two-way ANOVA with Dunnett's multiple comparisons (A), one-way ANOVA with Dunnett's multiple comparisons (B,C) or unpaired Student’s *t*-test (D) (**P<0.01, ****P<0.0001).

Mutations can impair homeostasis through effects on the gastrointestinal epithelium, the hematopoietic compartment, or both. To determine the relative contributions of hematopoietic and extrahematopoietic compartments, we generated bone marrow chimeric mice. Donors and/or recipients were either CD45.1 or *horton* (CD45.2) strains; chimeras were completely reconstituted with donor bone marrow as assessed by flow cytometry. After 1.4% DSS administration, chimeric CD45.1 mice with *horton* hematopoietic cells did not exhibit significant weight loss, similar to control CD45.1 mice receiving transplants of wild-type bone marrow (Fig. 3A). Chimeric *horton* recipient mice, irrespective of donor bone marrow, were not protected from DSS challenge, and lost an average of 25% of their initial body weight by day 8. The weight loss coincided with reduced colon length (Fig. 3B) and increased rectal bleeding and diarrhea (Fig. 3C). *Myo1d* expression was previously reported in the kidney and brain (Bahler et al., 1994). To determine whether increased DSS-water consumption contributed to the colitis phenotype, we challenged *Myo1d^htn/wpr^* mice with DSS by oral gavage. When treated with the same DSS dose, *Myo1d^+/+^* mice exhibited no weight loss, whereas *Myo1d^htn/wpr^* mice were sensitive to DSS administration and lost ∼25% of their initial body weight by day 8 (Fig. 3D). These data demonstrate that functional MYO1D in nonhematopoietic cells is necessary for restoration of intestinal homeostasis following DSS challenge.

### *Myo1d* mutant mice have normal intestinal differentiation

**Fig. 4.**
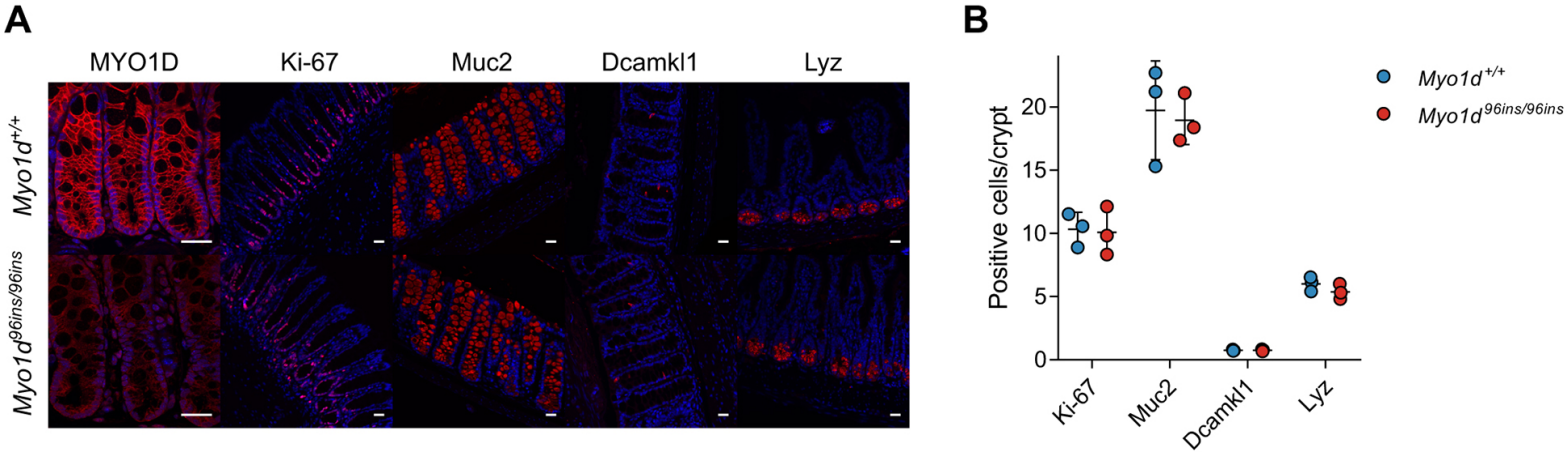
***Myo1d* mutant mice exhibit normal intestinal epithelial cell development.** (A) Representative images of immunofluorescence staining of ileum or colonic tissues for MYO1D, Ki-67, mucin 2 (Muc2), Dcamkl1 (also known as Dclk1), lysozyme (Lyz; also known as Lyz1) and nuclei in *Myo1d^+/+^* and *Myo1d^96ins/96ins^* mice. Scale bars: 25 μm. (B) Quantification of proliferating (Ki-67-positive), goblet (Muc2-positive), tuft (Dcamkl1-positive) and Paneth (Lyz-positive) cells in *Myo1d^+/+^* (*n*=3) and *Myo1d^96ins/96ins^* (*n*=3) animals.

In wild-type enterocytes of the small intestine, MYO1D localizes to the lateral membrane, terminal web and microvillar tips (Benesh et al., 2010). We found that in the colons of wild-type mice, MYO1D was chiefly produced by colonocytes, in which it was localized primarily to the basolateral membrane (Fig. 4A). In *Myo1d^96ins/96ins^* mice, it was either absent or mislocalized (Fig. 4A), suggesting that the mutant phenotype might result from an epithelial specific defect. Myosin IA (also known as Myosin 31DF), the *Drosophila* homolog of MYO1D, interacts with β-catenin and mutations in Myosin IA lead to defects in left-right asymmetry (Hozumi et al., 2006; Spéder et al., 2006). Wnt/β-catenin signaling maintains homeostasis of the intestinal epithelium by regulating the balance between cell proliferation, differentiation and death (Fevr et al., 2007). Therefore, we examined whether epithelial homeostasis was altered due to loss of MYO1D function without environmental insult. Similar numbers of proliferative cells [Ki-67 (also known as Mki67) positive], and goblet, Paneth and enteroendocrine cells were detected in *Myo1d^96ins/96ins^* and wild-type colon or ileum epithelium (Fig. 4A,B). These findings suggest that intestinal epithelial differentiation is normal in *Myo1d* mutant mice.

### MYO1D couples membrane lipids to actin filaments

**Fig. 5.**
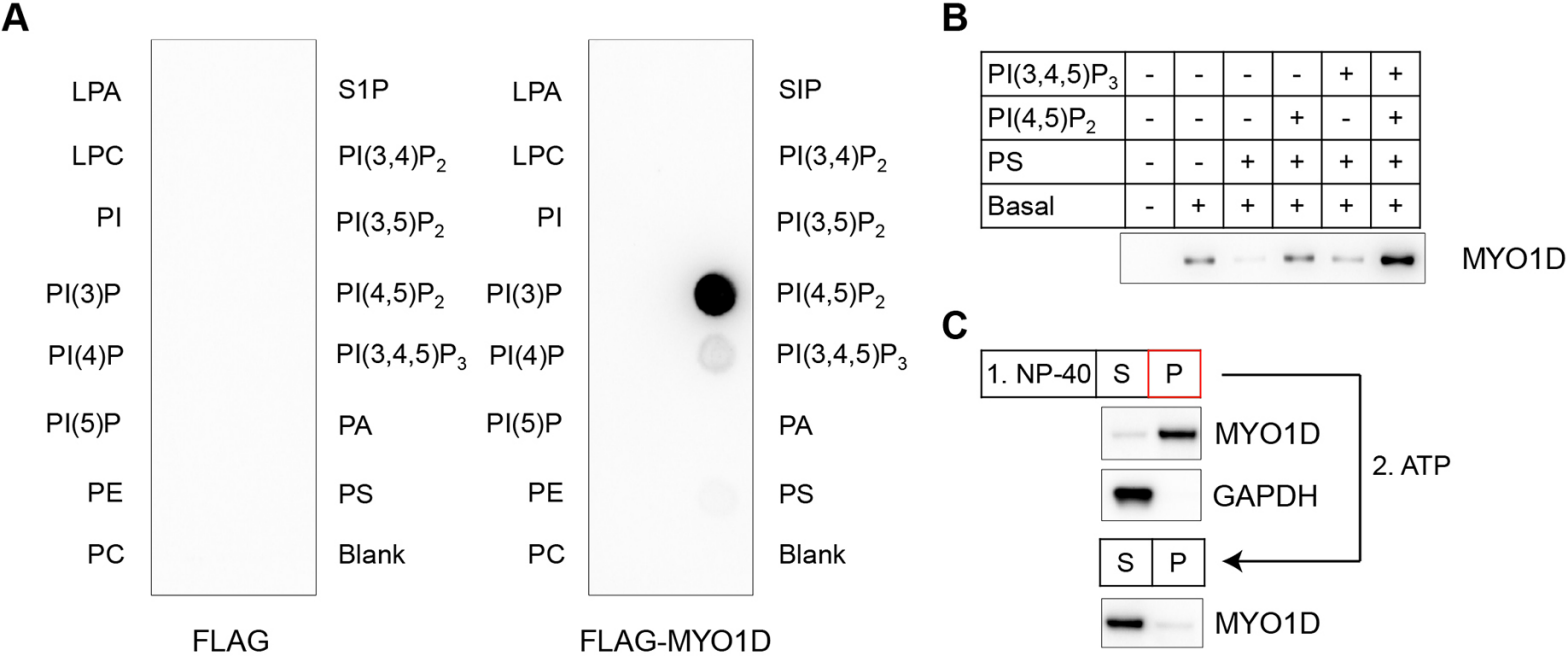
**Myosin 1D functions in actin and lipid binding.** (A) Representative images of FLAG-MYO1D bound to a lipid-coated strip membrane. FLAG alone bound to lipid-coated strip membrane was used as a negative control. (B) Representative immunoblot of the products of a liposome/protein co-flotation assay. Basal liposomes were composed of phosphatidylcholine, phosphatidylethanolamine, cholesterol and diacylglycerol. (C) Representative immunoblot of supernatant (S) and pellet (P) fractions of colonic epithelial cells lysed with 1% NP-40. The NP-40 pellet fraction was subsequently treated with 2 mM ATP in the absence of detergent. GAPDH was used to indicate solubilization of cytoplasm by 1% NP-40. LPA, lysophosphatidic acid; LPC, lysophosphocholine; PA, phosphatidic acid; PC, phosphatidylcholine; PE, phosphatidylethanolamine; PI, phosphatidylinositol; PI(3)P, PI-(3)-phosphate; PI(4)P, PI-(4)-phosphate; PI(5)P, PI-(5)-phosphate; PI(3,4)P_2_, PI-(3,4)-bisphosphate; PI(3,5)P_2_, PI-(3,5)-bisphosphate; PI(4,5)P_2_, PI-(4,5)-bisphosphate; PI(3,4,5)P_3_, PI-(3,4,5)-trisphosphate; PS, phosphatidylserine; S1P, sphingosine-1-phosphate.

To better understand the role of MYO1D in the epithelium, we investigated its biochemical properties. The TH1 domain of class I myosins is capable of binding lipid moieties (McConnell and Tyska, 2010). To determine the lipids that MYO1D binds, we purified recombinant human MYO1D, which shares 98% sequence identity with mouse MYO1D, and assessed binding to lipid strips (Fig. 5A). MYO1D exhibited strongest affinity for phosphatidylinositol 4,5-bisphosphate [PI(4,5)P_2_] and some binding to phosphatidylinositol (3,4,5)-trisphosphate [PI(3,4,5)P_3_]. To further assess the lipid binding of MYO1D, we performed a complementary liposome co-flotation assay (Fig. 5B). MYO1D showed highest co-flotation with liposomes containing both PI(4,5)P_2_ and PI(3,4,5)P_3_. The N-terminal motor domain of class I myosins mediates actin binding (McConnell and Tyska, 2010). To confirm the association of MYO1D with the actin cytoskeleton in the colon epithelium, we examined the association of MYO1D with the particulate fraction of NP-40-treated mouse colonic epithelial cell lysates (Fig. 5C). Under these conditions, the majority of MYO1D remained insoluble. Treatment of the insoluble pellet with 2 mM adenosine 5′-triphosphate (ATP) resulted in near complete solubilization of MYO1D, consistent with ATP disruption of the myosin-actin rigor conformation. These data suggest that MYO1D binds to PI(4,5)P_2_ or PI(3,4,5)P_3_, and to filamentous actin in intestinal epithelial cells.

## DISCUSSION

Using forward genetics, we demonstrated that damaging mutations in the class I myosin MYO1D led to a defect that rendered mice susceptible to DSS-induced colitis. Using multiple ENU alleles and CRISPR/Cas9 targeting, we validated causation. MYO1D appears to function as a molecular link between membrane lipids and the actin cytoskeleton.

The *whisper* phenotype originally mapped to three putative ENU-introduced mutations in *Myo1d*, *Ksr1* and *Lypd8*. Both *Ksr1* and *Lypd8* knockout (KO) mice are known to be susceptible to DSS-induced colitis (Goettel et al., 2011; Okumura et al., 2016). However, it is not clear that the alleles generated here confer susceptibility, and MYO1D was shown to be essential for resistance to DSS challenge by complementation testing, as well as gene targeting.

Functional studies of class I myosins in the intestinal epithelium have so far been limited to myosin 1A (MYO1A). MYO1A associates with brush border membrane rafts and is required for the localization or retention of sucrase-isomaltase and cystic fibrosis transmembrane conductance regulator channels (Kravtsov et al., 2012; Tyska and Mooseker, 2004). Additionally, MYO1A powers microvillar membranes to propel alkaline phosphatase-laden vesicles into the lumen (McConnell et al., 2009). *Myo1a* KO mice have many perturbations at the cellular level, but fail to show any phenotype at the level of the whole organism without environmental stress presumably because of compensation from other class I myosins including MYO1C and MYO1D (Tyska et al., 2005). *Myo1a* KO mice do exhibit increased mortality with 3% DSS treatment, but the death rate of the *Myo1d* mutant mice observed in response to 1.4% DSS in our study suggests that MYO1D has evolved a nonredundant function that is not compensated by other class I myosins.

The physiological function of MYO1D in the epithelium required to maintain intestinal homeostasis remains unknown, although several possibilities exist. One attractive hypothesis is that MYO1D regulates vesicle trafficking in the epithelium. In the epithelial Madin–Darby Canine Kidney cell line, MYO1D is required for trafficking of transferrin from apical and basolateral early endosomes to recycling endosomes (Huber et al., 2000). It is possible that MYO1D plays a crucial role in trafficking adhesion molecules to the basolateral membrane, where they are necessary for the integrity of adherens and tight junctions that are disrupted by DSS.

Class I myosins are well suited to regulate membrane dynamics owing to their ability to bind both actin and membrane lipids (McConnell and Tyska, 2010). Assessment of MYO1D binding properties suggests that the protein likely binds membrane lipids and actin *in vivo*. DSS treatment wounds the epithelium and requires cell migration into the damaged area for epithelial restitution. Remodeling of the actin cytoskeleton underlies the membrane dynamics necessary for cell migration and disruption of actin remodeling is sufficient to cause chemically-induced colitis in *Vil1*^−/−^ mice (Ferrary et al., 1999; Ubelmann et al., 2013). Collective cell migration also requires planar cell polarity, which is disrupted in rats lacking MYO1D (Hegan et al., 2015). Thus, impaired cell migration owing to disruption of actin cytoskeletal remodeling might also contribute to DSS-induced colitis susceptibility in *Myo1d* mutant mice.

The DSS-induced colitis model does not recapitulate all aspects of human IBD, eliciting a different profile of T cell proinflammatory cytokines, for example (Eichele and Kharbanda, 2017; Kiesler et al., 2015). However, DSS-induced colitis shares many similarities with human IBD, particularly with ulcerative colitis, including clinical manifestations and organ pathogenesis (Eichele and Kharbanda, 2017; Kiesler et al., 2015). Moreover, orthologs or paralogs of several genes linked to monogenic forms of human IBD have been implicated in DSS-induced colitis in mice by our laboratory (Brandl and Beutler, 2012; Brandl et al., 2010; Turer et al., 2017) and others (Bertolotti et al., 2001; Cox et al., 2012; Li et al., 2014; Staley et al., 2009; Watanabe et al., 2008), indicating that molecular mechanisms relevant to human IBD can be uncovered through study of the DSS model. The present study identified an essential and nonredundant role for MYO1D in restoring intestinal homeostasis following disruption of the epithelial barrier in mice, and should encourage consideration of MYO1D as a candidate locus in work seeking to identify genetic causes of IBD in humans.

## MATERIALS AND METHODS

### Mice

ENU mutagenesis was performed as previously described (Wang et al., 2015). For DSS-induced colitis induction, 8- to 12-week old mice received 1.3-1.5% (wt/vol) DSS in the drinking water for 7 days followed by 3 days without DSS. Mice gavaged with DSS received 2.5 g DSS/kg of bodyweight daily for 7 days via a gavage feeding needle. Body weight was recorded daily and reported as the amount of weight loss from the pre-treatment weight. Disease activity index score is a composite score of weight loss, stool bleeding and stool consistency, determined as previously described (Kim et al., 2012). Briefly, weight loss: 0 (no loss), 1 (1-10% loss of body weight), 2 (10-15% loss of body weight), 3 (15-20% loss of body weight) and 4 (>20% loss of body weight); stool consistency: 0 (normal), 2 (loose stool) and 4 (diarrhea); and bleeding: 0 (no blood), 1 (hemoccult positive), 2 (hemoccult positive and visual pellet bleeding) and 4 (gross bleeding and/or blood around anus). All mice were housed in the University of Texas Southwestern vivarium. All procedures were approved by the Institutional Animal Care and Use Committee of the University of Texas Southwestern Medical Center and were performed in accordance with institutionally approved protocols.

### Generation of the *Myo1d^96ins/96ins^* mouse strain using the CRISPR/Cas9 system

To generate the *Myo1d^96ins/96ins^* mouse strain, female C57BL/6J mice were superovulated by injection of 6.5 U pregnant mare serum gonadotropin (PMSG; Millipore), followed by injection of 6.5 U human chorionic gonadotropin (hCG; Sigma-Aldrich) 48 h later. The superovulated mice were subsequently mated overnight with C57BL/6J male mice. The following day, fertilized eggs were collected from the oviducts and *in vitro*-transcribed Cas9 mRNA (50 ng/μl) and *Myo1d* small base-pairing guide RNA (50 ng/μl; 5′-CCGTGCAGGCTGCACTGCACCGG-3′) were injected into the cytoplasm or pronucleus of the embryos. The injected embryos were cultured in M16 medium (Sigma-Aldrich) at 37°C in 5% CO_2_. For the production of mutant mice, two-cell stage embryos were transferred into the ampulla of the oviduct (10-20 embryos per oviduct) of pseudopregnant Hsd:ICR (CD-1) female mice (Harlan Laboratories). The resulting *Myo1d^96ins/96ins^* mice contain a 96 bp insertion (5′-ACAGAGAAGAAATGCACCGTCTCTGTGGAGACCCGGCTCAATCAGCCACAGCCTGACTTCACCAAGACCCGGCTCAATCAGCCACAGCCTGACTTC-3′) between nucleotides 80484343-80484342 on chromosome 11. The inserted sequence is composed of multiple duplication events from exon 22 of *Myo1d*. This insertion results in the in-frame addition of 32 amino acids (TEKKCTVSVETRLNQPQPDFTKTRLNQPQPDF) between residues 968 and 969 of the protein.

### Crypt isolation

Colonic crypts were isolated as previously described (Brandl et al., 2009). Briefly, colons were isolated from mice and stools removed from the lumen. Colons were then cut into 5-10 mm pieces and incubated at room temperature for 30 min in phosphate buffered saline (PBS) containing 5 mM EDTA.

### Antibodies

The following antibodies were used in this study: anti-GAPDH (1:1000; Cell Signaling Technology, D16H11), anti-Ki-67 (1:200; Cell Signaling Technology, D3B5), anti-Muc2 (1:200; Santa Cruz Biotechnology, H-300), anti-myosin ID [1:200 (immunofluorescence) or 1:1000 (western blotting); Santa Cruz Biotechnology, H-60], anti-lysozyme (1: 200; DAKO, 3.2.1.17) and anti-Dcamkl1 (1:300; Abcam, ab37994).

### Quantitative RT-PCR

Total RNA from colonic epithelium was isolated using TRIzol reagent (Thermo Fisher Scientific) according to the manufacturer's instructions. DNase treatment and clean-up was performed with a DNA-free DNase Treatment and Removal Reagents kit (Thermo Fisher Scientific). The isolated RNA was subsequently purified on a silica column (Invitrogen) to remove any excess DSS, which can interfere with the reverse transcriptase reaction. One microgram of RNA was reverse-transcribed to complementary DNA with SuperScript III First-Strand Synthesis System for RT-PCR (Life Technologies). Transcript levels of *Cxcl2*, *Ifng*, *Il1b*, *Il6*, *Nos2* and *Tnf* were analyzed using iTaq Universal SYBR Green Supermix (Bio-Rad) on a Step One Plus Real-Time PCR System (Life Technologies) with the following primers: *Cxcl2*: 5′-GCTTCCTCCTTCCTTCTGGT-3′, 5′-GGGCAGAAAGCTTGTCTCAA-3′; *Ifng*: 5′-TGAGCTCATTGAATGCTTGG-3′, 5′-ACAGCAAGGCGAAAAAGGAT-3′; *Il1b*: 5′-GGTCAAAGGTTTGGAAGCAG-3′, 5′-TGTGAAATGCCACCTTTTGA-3′; *Il6*: 5-GTCAGGGGTGGTTATTGCAT-3′, 5′-AGTGAGGAACAAGCCAGAGC-3′; *Nos2*: 5′- TTCTGTGCTGTCCCAGTGAG-3′, 5′-TGAAGAAAACCCCTTGTGCT-3′; and *Tnf*: 5′-AGATGATCTGACTGCCTGGG-3′, 5′-CTGCTGCACTTTGGAGTGAT-3′. Relative expression was calculated using the ΔΔCt standardization method using *Gapdh* (5′-TTGATGGCAACAATCTCCAC-3′ and 5′-CGTCCCGTAGACAAAATGGT-3′).

### Lipid strip binding assay

HEK293T cells (ATCC CRL-11268, authenticated and used between passages 5-10) overexpressing human FLAG-MYO1D were lysed in Nonidet P-40 buffer [50 mM Tris-HCl, pH 8.0, 0.1 M NaCl, 1% (vol/vol) Nonidet P-40, 10% (vol/vol) glycerol, 1.5 mM EDTA and Protease Inhibitor Mixture]. Immunoprecipitation was performed using anti-FLAG M2 agarose (Sigma-Aldrich). Binding of purified FLAG-MYO1D to lipid was analyzed using commercially available lipid strips (Echelon Biosciences) according to the manufacturer's instructions.

### Liposome co-flotation assay

Phosphatidylcholine (PC), phosphatidylethanolamine (PE), phosphatidylserine (PS), diacylglycerol (DAG), cholesterol (Chol), PI-(4,5)-bisphosphate [PI(4,5)P_2_] and PI-(3,4,5)-trisphosphate [PI(3,4,5)P_3_] were purchased from Avanti Polar Lipid. Liposomes were composed of PC:PE:Chol:DAG (58:20:20:2), PC:PE:Chol:DAG:PS (40:20:20:2:18), PC:PE:Chol:DAG:PS:PI(4,5)P_2_ (38:20:20:2:18:2), PC:PE:Chol:DAG:PS:PI(3,4,5)P_3_ (38:20:20:2:18:2) or PC:PE:Chol:DAG:PS:PI(4,5)P_2_:PI(3,4,5)P_3_ (36:20:20:2:18:2:2). Lipid mixtures were dried in glass tubes with nitrogen gas and under vacuum overnight followed by hydration in Tris-buffered saline (TBS, pH 7.6). Liposomes were prepared by extrusion 30 times through a 100-nm polycarbonate membrane. MYO1D protein was purified as described above. Liposome protein mixtures were incubated at room temperature for 1 h. Liposomes were isolated by flotation on a Histodenz density gradient (40:35:30%) as described and analyzed for binding to MYO1D by western blotting.

### Immunofluorescence

Intestine was rapidly dissected and flushed with cold PBS, cut into small 3 mM concentric circles, and fixed in freshly prepared 4% paraformaldehyde for 1 h at room temperature. Tissue was cryoprotected in 15% sucrose for 1 h and 30% sucrose overnight. Tissues were embedded in liquid nitrogen-cooled isopentane in optimum cutting temperature (OCT) compound in the same mold to ensure identical processing for each sample. Sections (7 μm) were produced using a Leica cryostat. The sections were washed with PBS 3× for 5 min, and then blocked in 10% bovine serum albumin (BSA) in PBS. Primary antibodies were diluted in 5% BSA in PBS. Sections were incubated overnight at 4°C and then washed 3× for 10 min. Slides were then incubated for 1 h in Alexa Fluor dye-conjugated antibodies (1:1000) in 5% BSA in PBS and washed 3× for 10 min. Slides were mounted in Prolong Antifade Gold and visualized using a Zeiss LSM880 confocal microscope.

### Statistical analysis

Age- and sex-matched mice were randomly allocated to experimental groups based on their genotypes. No pre-specified effect size was assumed, and three to 12 mice per genotype were used in experiments; this sample size was sufficient to demonstrate statistically significant differences in comparisons between two or more unpaired experimental groups by unpaired Student's *t*-test or ANOVA, respectively. All mice were included during data analysis. All statistical analyses were performed using GraphPad Prism. Two-tailed Student's *t*-test was utilized for comparisons of a single parameter between two groups, one-way ANOVA with Dunnett's test was utilized for comparison of one parameter between multiple groups, and two-way ANOVA with Dunnett's test was utilized for comparison of two parameters between multiple groups. Because mice utilized in this study were inbred and age- and sex-matched, variance was assumed to be similar between treatment groups. Phenotypic data were assumed to follow a normal distribution, as has been observed in large datasets from numerous phenotypic screens conducted by our group.
